# Association Between Optimism and Incident Stroke Among Stroke Survivors: Findings From the English Longitudinal Study of Ageing

**DOI:** 10.1093/abm/kaad051

**Published:** 2023-09-14

**Authors:** Joseph Chilcot, Ruth A Hackett

**Affiliations:** Health Psychology Section, Department of Psychology, Institute of Psychiatry, Psychology and Neuroscience King’s College London, London, UK; Health Psychology Section, Department of Psychology, Institute of Psychiatry, Psychology and Neuroscience King’s College London, London, UK

**Keywords:** Personality, Stroke, Health behavior, Big-five

## Abstract

**Background:**

Personality has been implicated in stroke death. However, the role of personality in stroke incidence is unclear.

**Purpose:**

Our primary aim was to investigate associations between optimism, determination, control, and the “Big Five” personality traits on incident stroke. A secondary aim was to assess the potential mediating role of health behaviors in the personality-stroke relationship.

**Methods:**

A total of 3,703 stroke-free participants from the English Longitudinal Study of Ageing provided data on personality using the Midlife Development Inventory at Wave 5 (2010/11). Self-reported incident stroke was assessed from Waves 6 to 8 (2012–2017). Associations were modeled using discrete-time survival proportional odds logistic models. Analyses were adjusted for sociodemographic factors, history of other cardiometabolic diseases, and health behaviors.

**Results:**

Over 6 years follow-up there were 125 incident strokes. Higher optimism (hazard ratio [HR] = 0.66; 95% confidence interval [CI] 0.53, 0.82), openness (HR = 0.72; 95% CI 0.53, 0.98), and conscientiousness (HR = 0.59; 95% CI 0.42, 0.84) were associated with reduced incident stroke risk in unadjusted models. After adjustment for sociodemographic factors and history of cardiometabolic disease, only the association between optimism and incident stroke remained significant (HR = 0.72; 95% CI 0.57, 0.92). The effect of optimism remained significant in a final model adjusting for health behaviors (HR = 0.75; 95% CI 0.60, 0.96). There was evidence of a small but significant mediating effect of physical activity.

**Conclusions:**

Higher trait optimism was associated with reduced stroke risk. This association was partially mediated by physical activity albeit the effect was small, and caution warranted inferring causality. The interplay of personality, behavior, and clinical risk factors in stroke incidence and survivorship needs further investigation.

## Teaser

Coronary heart disease (CHD) patients who had high levels of distress overtime were likely to hold more negative and unhelpful illness perceptions, including weaker control related beliefs, compared to those with stable-low distress symptoms.

## Introduction

Around 10% of global deaths are attributable to stroke [1], which is one of the leading causes of acquired disability [2, 3]. Approximately 90% of stroke risk is accounted for by 10, potentially modifiable factors, including hypertension, physical activity, smoking status, alcohol use, and depression [4]. Many cases of stroke are therefore potentially preventable through the control of metabolic risk factors and behavior change [5].

The five-factor model of personality (the “Big-Five”) [6] describes five traits: *Neuroticism* (the proclivity to experience distress and negative emotions), *Extraversion* (the tendency to experience positive emotions and be sociable), *Openness* (the tendency to be curious and open to new experiences), *Agreeableness* (the tendency to be cooperative), and *Conscientiousness* (the proclivity to be organized and self-disciplined). There is consistent evidence showing that aspects of personality are associated with well-being and health outcomes, including mortality [7–12], disease progression in HIV [13], carotid atherosclerosis [14], and wound healing [15, 16]. Meta-analytic evidence has found a robust association between neuroticism and incident Parkinson’s disease [17] and dementia [18].

Dispositional optimism (the tendency to have positive expectations about the future) is closely associated with four of the Big-Five traits, with greater optimism related to higher emotional stability, extraversion, agreeableness, and conscientiousness [19]. Higher optimism has been associated with a lower risk of nonfatal myocardial infarction among older community-dwelling adults [20]. The multiple mechanisms through which personality exerts effects on health and well-being are complex [21, 22]. The health-behavior model of personality [23, 24] posits that personality traits influence health outcomes through engagement with health-promoting or health-compromising behaviors, including seeking medical consultation, smoking, and physical activity [25–27]. For example, smoking has been found to partially mediate the association between neuroticism and mortality [28]. In another study, drinking, smoking, and waist circumference mediated the association between conscientiousness and mortality, by 42% [27]. In addition to the indirect behavioral pathway linking personality and health, another possibility is that personality may impact health outcomes through direct biological pathways. For example, optimism is suggested to exert a protective influence on cardiovascular stress responses in both healthy [29] and clinical samples [30]. In turn, pooled evidence has associated dysregulated cardiovascular stress responses with incident cardiac events [31]. Changes in diurnal cortisol output (an indicator of hypothalamic pituitary adrenal [HPA] axis activity) have also been associated with optimism [30]. Data from large observational studies suggest that disturbances in the patterning of daily cortisol output are associated with an increased risk of cardiometabolic mortality and morbidity [32, 33].

While there is growing evidence for the personality-health outcome association, we are not aware of any studies testing the relationship between personality and incident stroke. There is evidence showing that higher extraversion is associated with an increased risk of stroke mortality, while higher conscientiousness is associated with reduced stroke mortality risk [34]. The primary aim of this study was to test the association between personality and incident stroke risk using data from the English Longitudinal Study of Aging (ELSA) [35]. The secondary aim was to explore if health-related behaviors mediated any associations between personality and incident stroke. Given the current evidence base, we hypothesized that higher levels of optimism and conscientiousness would reduce the risk of incident stroke, with greater extraversion and neuroticism associated with increased risk. Furthermore, we predicted that health behaviors would partially mediate any observed personality-stroke risk association.

## Methods

### Participants

Data were from the ELSA [35–37], a representative prospective panel study of people living in England aged 50 years and older. The initial ELSA sample was drawn from the Health Survey for England, a yearly cross-sectional survey designed to monitor the health of the general population. The sociodemographic profile of the sample is considered representative of the English population (based on a comparison with findings from the Census). Participants provide data on health, well-being, economic, and social circumstances. ELSA began in 2002/3 (Wave 1), following participants up every 24 months. At each study wave, interview and questionnaire data are collected. At alternate study waves, anthropometric and biological data is collected in person during a nurse visit. The present study uses data gathered in Wave 5 (2010/11), where measures of personality were included. Incident stroke was assessed over Waves 6–8 (2012/13 to 2016/17). Ethical approval for ELSA was obtained from the National Research Ethics Service in accordance with the Declaration of the World Medical Association. All participants gave full informed consent.

A total of 10,274 participants took part in Wave 5 of ELSA. Participants were included in the present study if they completed the personality measures and had no documented history of stroke. A sample of 5,250 people with no history of stroke between Waves 1 and 5 was initially identified (373 had a history of stroke during Waves 1–5). Of these 4,334 provided information on personality and stroke. Of these, 4,015 had data on sociodemographic and clinical characteristics. As our secondary aim was to investigate the role of health behavior in the personality-stroke link, we further restricted the sample to those who provided complete information on health behavior, resulting in an analytic sample of 3,703 participants.

In comparison to those excluded from the analysis (*n* = 6,335), those included reported lower neuroticism and had higher levels of optimism and control (*p* < .05). No other significant differences in personality were observed. In terms of sociodemographic characteristics, the retained sample was significantly older, more likely to be of white ethnicity and wealthier (*p* < .001) than the excluded sample. They were also less likely to meet the cutoff for depressive symptoms (*p* < .001). The groups did not significantly differ in sex, marital status, or history of cardiometabolic disease. Focusing on health behavior, the analytic sample was less likely to smoke and to be inactive (*p* < .001) than the excluded sample. No significant differences in alcohol consumption were observed.

## Measures

### Personality

Personality traits were measured using a modified version of the Midlife Development Inventory (MIDI) [38]. The MIDI measures the Big-Five personality traits [6] using 26 adjectives rated on a four-point scale: Neuroticism (four items), Extraversion (five items), Openness (seven items), Conscientiousness (five items), and Agreeableness (five items). Higher scores on all scales indicate higher levels of the trait (e.g., high conscientiousness). In addition to the MIDI, optimism, determination, and control were also measured as related aspects of personality as in previous ELSA studies [39]. Optimism was measured with the average ratings of two statements *“I feel that life is full of opportunities”* and *“I feel that the future looks good for me,”* scored on a four-point scale. Determination was assessed using a single-item rating of the extent to which participants had felt determined over the past 30 days (responses ranged from "not at all" to "very much" on a five-point scale). Control was measured by a single item “*At home, I feel I have control over what happens in most situations,”* on a six-point scale. Higher scores on these scales indicate greater levels of the trait. Cronbach’s alpha and omega reliability coefficients for each scale are presented in the results section.

### Primary Outcome

Participants were asked whether a physician had given them a diagnosis of stroke since their last interview. Incident stroke across Waves 6–8 (2012/13 to 2016/17) was the primary outcome. The event observation period was 72 months (interval time between Waves 5 and 8).

### Covariates

Discrete-time survival proportional odds logistic models were adjusted for covariates at Wave 5 (2010/2011) thought to be associated with personality and stroke risk. Initial model adjustments included age (in years), sex (male/female), marital status and ethnicity (white vs. other), household nonpension wealth (quintiles, as used previously [37]), self-reported diabetes, and coronary heart disease. As used in previous ELSA analyses [39–41] depressive symptoms were evaluated using the 8-item version of Center for Epidemiologic Studies Depression Scale (CES-D) [42], with a score of ≥4 indicating higher symptoms and possible depression. Additional models were adjusted for health behaviors: smoking status (current smoker, yes vs. no), physical activity level (using a 5-level categorization; sedentary—active as defined elsewhere [43], and treated as an ordinal variable here), and the number of units of alcohol consumed during the past week on the heaviest consumption day.

### Statistical Analysis

Descriptive characteristics of the sample are presented as either mean (standard deviation) or number (percentage). Associations between personality traits and health behaviors were explored using Spearman’s rho (ρ) correlations (alcohol units and physical activity) and independent samples *t*-tests (smoking status). Incident stroke was evaluated over Waves 6 - 8 with right censoring. Since we were observing stroke events that occurred during intervals (study waves), discrete-time survival proportional odds logistic models with Maximum Likelihood estimation (ML) were used, using the MPlus “DSURVIVAL” command. A latent survival hazard function (f) was specified using “F by w6stroke_new-w8stroke_new@1; F@0;.” These models were used to estimate hazard ratios (HRs) by exponentiating model estimates, with 95% confidence intervals (CI), per unit change in personality (these exponentiated estimates are more closely aligned to a hazard rather than an odds ratio, although some refer to these as *hazard-odds ratios*). Therefore, due to the logistic model, the HRs estimated here have a similar interpretation, but are technically different, to HRs that are estimated in Cox-regression models.

Our secondary aim was to explore if health behaviors mediated any associations between personality and incident stroke, using mediation structural equation discrete-time survival models, with ML estimation and bootstrapped (5,000 resamples) standard errors and 95% CI for path estimates. These estimated simultaneous total, direct and indirect effects of optimism and health behavior on incident stroke, and the portion of the total effect accounted for by the indirect effects. Since this was an exploratory analysis, a simple mediation approach, which included the addition of covariates, was undertaken [44]. This approach assumes a causal variable (optimism) is associated with the outcome (latent survival function) and is correlated with the mediator (health behavior) which in turn affects the outcome; assumptions that were supported. More advanced model-based approaches typically assume temporal separation between the independent variable and mediator, which was not possible here due to the study design. Furthermore, although the effect of only one health behavior (physical activity) was correlated with optimism and associated with the latent survival structure, we decided to also include direct and indirect paths for alcohol and smoking status since these were related to physical activity levels. Removing these two health behaviors from the model did not change the results reported below. Analyses were conducted using SPSS version 27 [45] and MPlus version 8.8 [46].

## Results

Sample demographics at Wave 5 are shown in Table 1 (*n* = 3,703). Descriptive statistics (means), reliabilities, and correlations between the personality traits are shown in Table 2. All personality scales had adequate internal reliability.

**Table 1:** Sample characteristics (*n* = 3,703)

Characteristic	Statistic
Age (years)	69.1 (7.9)
Sex (% female)	2,086 (56.3)
Married (% yes)	2,495 (67.4)
Wealth (£)	
Quintile 1	504 (13.6)
Quintile 2	703 (19.0)
Quintile 3	780 (21.1)
Quintile 4	824 (22.3)
Quintile 5	892 (24.1)
Ethnicity (% white)	3,653 (98.6)
Smoker	
Never	3,224 (87.1)
Ex-smoker	117 (3.2)
Current	362 (9.8)
Physical activity level	
Sedentary (level 0)	523 (14.1)
Active (level 5)	696 (18.8)
Alcohol units^a^	2.72 (5.6)
CHD (% yes)	499 (13.5)
Diabetes (% yes)	417 (11.3)
CESD- case (% yes)^b^	427 (11.5)

Data are presented as mean (standard deviation) or number (percentage).

*CES-D* Center for Epidemiologic Studies Depression Scale score; *CHD* coronary heart disease.

^a^On heaviest drink day in past week.

^b^Scores >4 cutoff for likely depression.

**Table 2:** Descriptive statistics and correlations between personality traits (*n* = 3,703)

	Mean (*SD*)	Reliability ^a^	1	2	3	4	5	6	7
1.Optimism	3.11 (0.73)	0.78^b^	–						
2.Determination	3.67 (0.96)	–	0.487**	–					
3.Control	5.22 (0.90)	–	0.376**	0.281**	–				
4.Neuroticism	2.05 (0.57)	0.69	−0.274**	−0.194**	−0.265**	–			
5.Extraversion	3.14 (0.55)	0.75	0.468**	0.485**	0.299**	−0.197**	–		
6.Openness	2.86 (0.55)	0.79	0.411**	0.441**	0.218**	−0.166**	0.594**	–	
7.Agreeableness	3.50 (0.48)	0.80	0.238**	0.273**	0.175**	−0.031	0.553**	0.407**	–
8.Conscientiousness	3.28 (0.49)	0.69	0.347**	0.391**	0.236**	−0.195**	0.473**	0.458**	0.431**

*SD* standard deviation.

^a^McDonald’s Omega.

^b^Cronbach’s Alpha. Omega cannot be calculated with only two items.

Determination and control had single items, so reliability coefficients cannot be calculated.

**p < .01.

### Association Between Personality and Health Behaviors

The number of reported units of alcohol consumed on the heaviest drinking day in the last week showed small but significant negative associations with neuroticism (Spearman’s ρ = −0.05, *p* < .01) and agreeableness (Spearman’s ρ = −0.09, *p* < .01); and positive associations with extraversion (Spearman’s ρ = 0.04, *p* < .01), openness (Spearman’s ρ = 0.10, *p* < .01), control (Spearman’s ρ = 0.57, *p* < .01), and optimism (Spearman’s ρ = 0.08, *p* < .01).

Smoking status was significantly associated with optimism (mean difference = 0.26, *t* = 6.55, *df* = 3,701, *p* < .001) and agreeableness (mean difference = 0.07, *t* = 2.38, *df* = 3,701, *p* < .01), with current smokers having significantly lower optimism, but higher agreeableness, albeit the effects were very small. Higher physical activity (treated as an ordinal variable) was positivity associated with extraversion (Spearman’s ρ = 0.213, *p* < .01), openness (Spearman’s ρ = 0.177, *p* < .01), conscientiousness (Spearman’s ρ = 0.176, *p* < .01), optimism (Spearman’s ρ = 0.268, *p* < .01), determination (Spearman’s ρ = 0.181, *p* < .01), and control (Spearman’s ρ = 0.174, *p* < .01). There was a negative association between physical activity and neuroticism (Spearman’s ρ = −0.59, *p* < .01).

### Discrete-Time Survival Proportional Odds Logistic Models: Incidence Stroke

Over Waves 6–8 there were 125 (3.4%) incident stroke events (see Table 3 for proportions at each wave). Table 4 shows the unadjusted and adjusted associations between personality and incident stroke. In unadjusted models, higher levels of optimism (HR = 0.66 [95% CI 0.53, 0.82], *p* < .01), conscientiousness (HR = 0.59 [95% CI 0.42, 0.84], *p* < .01), and openness (HR = 0.72 [95% CI 0.53, 0.98], *p* < .05) were associated with a reduction in the hazard of incident stroke over Waves 6–8. After adjustment for sociodemographic and clinical factors, only the association between optimism and stroke remained significant (HR = 0.72 [95% CI 0.57, 0.92], *p* < .01), where a one-unit increase in optimism was associated with a 28% reduction in the hazard of incident stroke across the study intervals. This association remained significant after further adjustment for health behaviors (HR = 0.75 [95% CI 0.60, 0.96], *p* < .05). In this model the only health behavior significantly associated with stroke was physical activity (HR = 0.85 [95% CI 0.74, 0.98], *p* < .05). The minimal detectable effect size of optimism in adjusted Model 3 with preserved 80% power was estimated to be a HR of 0.68.

**Table 3:** Proportion of stroke incidence per study wave

	Wave 6	Wave 7	Wave 8
Number of events	37	40	48
Incidence rate (%)	1	1.2	1.7
Probability incidence rate (%)	1	1.3	2.0

**Table 4:** Unadjusted and adjusted associations between personality and incident stroke (*n* = 3,703)

Personality trait	Hazard Ratios (95% confidence intervals)		
	Model 1: Unadjusted	Model 2: Adjusted^a^	Model 3: Health behavior adjusted^b^
Optimism	0.66 (0.53, 0.82)**	0.72 (0.57, 0.92)**	0.75 (0.60, 0.96)*
Determination	0.91 (0.76, 1.10)	0.95 (0.79, 1.15)	0.98 (0.81, 1.19)
Control	0.87 (0.72, 1.05)	0.88 (0.73, 1.07)	0.89 (0.74, 1.09)
Big-Five			
Neuroticism	1.21 (0.81, 1.57)	1.37 (0.95, 1.98)	1.38 (0.96, 2.00)
Extraversion	0.82 (0.59, 1.13)	0.96 (0.69, 1.36)	1.03 (0.72, 1.47)
Openness	0.72 (0.53, 0.98)*	0.80 (0.58, 1.08)	0.83 (0.61, 1.14)
Agreeableness	0.93 (0.64, 1.34)	1.01 (0.68, 1.48)	1.00 (0.68, 1.48)
Conscientiousness	0.59 (0.42, 0.84)**	0.70 (0.48, 1.02)	0.74 (0.51, 1.07)

^a^Models adjusted for age, sex, marital status, ethnicity, nonpension wealth quintiles, presence of coronary heart disease, presence of diabetes, and depression (CES-D score >4).

^b^Models adjusted for all covariates in Model 2 and health behaviors (smoking status, physical activity and alcohol units).

**p ≤ .01, *p < .05.

### Indirect of Health Behavior on Stroke

**Fig. 1.**
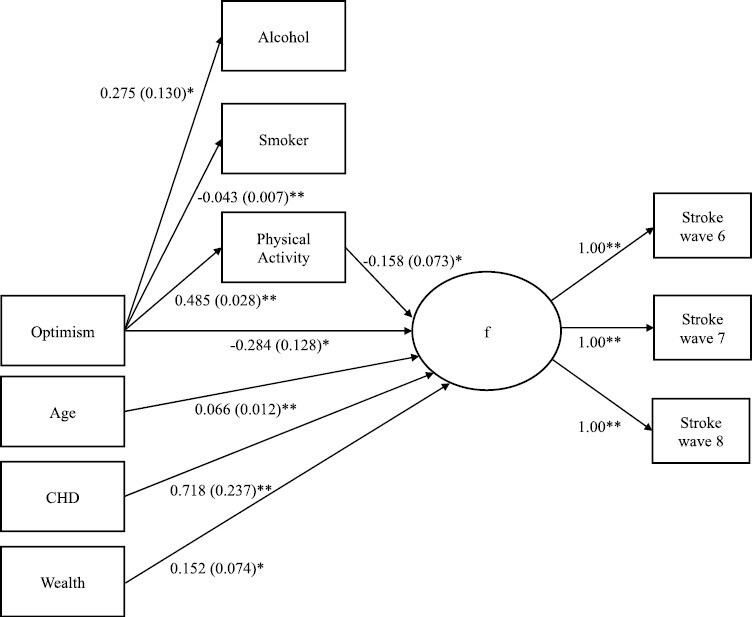
Mediation structural equation discrete-time survival models for the effect of optimism on stroke risk via health behaviors. Only significant paths shown. *F* = latent survival hazard. Model estimates shown with bootstrapped standard errors. **p < .01, *p < .05. CHD= Coronary Heart Disease.

Total, direct and indirect estimates of the effects of optimism and health behavior on incident stroke are shown in Table 5. Figure 1 displays the bootstrapped structural model tested. In this model, physical activity was predicted by optimism (estimate = 0.485, 95% CI = 0.431, 0.539). Incident stroke (survival function, f) was predicted by physical activity (estimate = −0.158, 95% CI = −0.300, −0.016) and optimism (estimate = −0.284, 95% CI = −0.534, −0.034). Overall, there was a small but significant total indirect effect of health behavior (estimate = −0.098, 95% CI = −0.171, −0.026), which accounted for approximately 25% of the total effect from optimism to stroke. Of the specific indirect effects, only physical activity was significant (estimate = −0.077, 95% CI = −0.146, −0.008), suggesting that the effect of optimism on incidence stroke was partially mediated by physical activity levels. Physical activity accounted for approximately 20% of the total effect of optimism on incident stroke. The minimal detectable indirect effect coefficient was −0.032, given preserved power of 80%.

**Table 5:** Total and indirect estimates of the effect of optimism and health behavior on stroke

	Estimate	*SE*	*p*-value
Effect from optimism to stroke			
Total	−0.382	0.130	0.003
Total indirect	−0.098	0.037	0.008
Indirect effect via physical activity	−0.077	0.035	0.030
Indirect effect via alcohol	0.001	0.006	0.899
Indirect effect via smoking	−0.022	0.014	0.099
Direct effect of optimism	−0.284	0.128	0.026

*SE* standard error.

## Discussion

The aim of this study was to test the association between personality and incident stroke risk among stroke survivors, using data from a community-dwelling sample of middle-aged and older adults from the ELSA study [35]. Furthermore, given the association between personality and behavior, we examined whether health-related behaviors attenuated any observed associations.

After adjustment for sociodemographic and clinical factors, higher levels of optimism appeared to be protective against incident stroke. This effect was maintained after adjustment for health behaviors. Conscientiousness and openness were only associated with incident stroke risk in unadjusted models, and we observed no effect of extraversion or neuroticism, therefore partially rejecting our first hypotheses. In exploratory mediation analysis, our second hypothesis was partially supported. We observed a significant indirect effect of physical activity, suggesting that some of the association between higher optimism and reduced stroke risk might be partly accountable by greater physical activity.

These findings support the growing evidence base for personality as a predictor of health outcomes and well-being [7–11]. Past studies have also shown that lower optimism is associated with poorer outcomes including greater disease progression in people with carotid atherosclerosis [14] and poorer wound healing in the general population [47] and surgical samples [16]. Our findings also align with past research showing a relationship between personality and health-related behaviors [25–27]. We found that trait optimism was lower in current smokers when compared with nonsmokers. Furthermore, physical activity was associated with all personality traits examined here, except for agreeableness. This provides some support for the theoretical health-behavior of personality model [23, 24] which posits that personality traits influence health-related outcomes through health-promoting or health-compromising behaviors.

Low physical activity is a recognized independent risk for stroke [48], and health behaviors such as physical activity are amenable to change. There is evidence that physical activity interventions may be efficacious in reducing stroke risk factors [49]. However, research on the efficacy of such interventions on incident stroke is lacking, mostly due to the length of follow-up required to observe any effects.

The strengths of the current study include the use of a large nationally representative sample of middle-aged and older adults living in England. We were able to consider several sociodemographic, behavioral, and clinical factors in our analyses. Additionally, our findings linking optimism with incident stroke were robust to adjustment for depression, a known stroke risk factor [50]. This strengthens the argument that positive affect is not merely the inverse or the absence of negative affect [51] and has an independent impact on cardiometabolic health [52].

When considering our findings, limitations must also be acknowledged. While we detected a novel association between optimism and incident stroke amongst stroke survivors, our models were not adjusted for anthropometric and biological marks indicated in stroke, as these were not collected at Wave 5. Furthermore, in addition to optimism, different aspects of positive well-being (such as enjoyment or purpose in life) may differentially impact health outcomes [53]. Future work should investigate whether associations with incident stroke vary across types of well-being. In addition, personality was only assessed at one point in time, therefore the influence of possible change in personality over time was not considered. However, previous work indicates that personality is relatively stable in adulthood [54]. Our mediation analysis should be considered exploratory, and caution taken inferring possible causality given our simple mediation approach and lack of temporal separation between the measurement of personality and health behavior.

Our main outcome was participant-reported stroke, therefore our findings are limited to stroke survivors and not stroke-related mortality or overall incidence risk. Therefore, it is important to recognize these data relate to an association between personality and survivable stroke incidence. It is possible that these findings point to an association between personality and stroke survivorship, albeit the limitations of the study prevent any firm interpretation of this since we do not have data on fatal stroke incidence. Further studies should examine the association between personality with incident fatal and nonfatal stroke using a competing risk approach to better understand how personality might be associated with stroke-related events and outcomes.

A further limitation is that we did not have information on stroke subtypes and severity. The use of clinically derived stroke diagnoses may have garnered different results, though a high concordance between self-reported and clinical stroke diagnoses has been reported previously [55]. Additionally, stroke incidence varies by ethnic group [56]. Since ELSA has a small number of ethnic minority participants, our findings may not generalize to these groups. We also excluded participants with missing data; those who were included reported lower neuroticism and had higher levels of optimism and control, were significantly older, more likely to be of white ethnicity and wealthier, and were less likely to meet the cutoff for depressive symptoms. They were also less likely to be inactive or smoke, meaning selection bias due to nonrandom exclusion is possible. This may limit the generalizability of our findings. We found significant associations between personality and health behavior but did not consider diet. Investigating the links between personality and nutrition would be beneficial in future work. Furthermore, health behaviors were self-reported, so future research should assess whether objective measures of physical activity play a role in the optimism-stroke relationship. Lastly, some of the associations between personality and health behavior (e.g., smokers being more agreeable), are in an unexpected direction, albeit these associations were small and may be spurious. Importantly, the associations between personality and physical activity were consistent with what would be expected.

In conclusion, in this study investigating the link between personality and incident stroke, we observed that higher trait optimism was associated with a reduced risk of stroke in those who survived, over 6 years follow-up. This association was independent of sociodemographic factors and history of cardiometabolic disease. This association was partially mediated by physical activity, albeit the effect was small, and caution is warranted inferring causality. The complex interplay of personality, behavior, and clinical risk factors in stroke risk and stroke survivorship should be further investigated in future studies.
